# Suppression of Excessive Histone Deacetylases Activity in Diabetic Hearts Attenuates Myocardial Ischemia/Reperfusion Injury via Mitochondria Apoptosis Pathway

**DOI:** 10.1155/2017/8208065

**Published:** 2017-01-16

**Authors:** Yang Wu, Yan Leng, Qingtao Meng, Rui Xue, Bo Zhao, Liying Zhan, Zhongyuan Xia

**Affiliations:** Department of Anesthesiology, Renmin Hospital of Wuhan University, Wuhan, Hubei Province 430060, China

## Abstract

*Background*. Histone deacetylases (HDACs) play a pivotal role in signaling modification and gene transcriptional regulation that are essential for cardiovascular pathophysiology. Diabetic hearts with higher HDACs activity were more vulnerable to myocardial ischemia/reperfusion (MI/R) injury compared with nondiabetic hearts. We are curious about whether suppression of excessive HDACs activity in diabetic heart protects against MI/R injury.* Methods*. Diabetic rats were subjected to 45 min of ischemia, followed by 3 h of reperfusion. H9C2 cardiomyocytes were exposed to high glucose for 24 h, followed by 4 h of hypoxia and 2 h of reoxygenation (H/R).* Results*. Both MI/R injury and diabetes mellitus elevated myocardium HDACs activity. MI/R induced apoptotic cell death was significantly decreased in diabetic rats treated with HDACs inhibitor trichostatin A (TSA). TSA administration markedly moderated dissipation of mitochondrial membrane potential, protected the integrity of mitochondrial permeability transition pore (mPTP), and decreased cell apoptosis. Notably, cotreatment with Akt inhibitor partly or absolutely inhibited the protective effect of TSA in vivo and in vitro. Furthermore, TSA administration activated Akt/Foxo3a pathway, leading to Foxo3a cytoplasm translocation and attenuation proapoptosis protein Bim expression.* Conclusions*. Both diabetes mellitus and MI/R injury increased cardiac HDACs activity. Suppression of HDACs activity triggered protective effects against MI/R and H/R injury under hyperglycemia conditions through Akt-modulated mitochondrial apoptotic pathways via Foxo3a/Bim.

## 1. Introduction

Histone deacetylases (HDACs) and the histone acetyl transferases (HATs) regulated global acetylation levels in mammals. They keep a delicate balance and mainly function through acetylation/deacetylation of histone and nonhistone substrates [1]. HDACs family is composed of three distinct groups, nonsirtuin HDACs (class I, II) and longevity protein sirtuin deacetylases (class III) [2]. Previous analyses have grouped nonsirtuin HDACs into two different classes, based on DNA sequence similarity and function [3]. Class I HDACs (1, 2, 3, and 8) display an extensive presence in nucleus and exhibit tissue-specific distributions [4]. Class II HDACs (4, 5, 6, 7, 9, and 10) can shuttle between the nucleus and cytoplasm [5]. Class I HDACs have been extensively studied in their classical role as histone modifiers and transcriptional repressors [6, 7]. Class II HDACs primarily control gene expression by recruiting other proteins; in some cases, these enzymes can also act as transcriptional activators [8]. Many studies indicated that acetylation levels of mammal are linked strongly with cardiovascular diseases, including coronary heart diseases [9], diabetic cardiomyopathy [10], hypertension [11], ventricular remodeling [12, 13], and arrhythmia [14]. Recently, selective inhibition of classes I and II HDACs with an inhibitor, trichostatin A (TSA), showed protective effects against MI/R injury [15]. This is in line with the observations that inhibition of HDACs in cardiac myocytes silences fetal gene activation, attenuates cardiac hypertrophy, and prevents cardiac remodeling [16, 17]. Furthermore, HDACs inhibition was recently reported to improve myocardial function and prevent cardiac remodeling in diabetic mice [18].

Epidemiological surveys indicated that diabetic patients are more likely to develop cardiovascular complications [19]. Despite current optimal therapy, the mortality rate of acute myocardial infarction in diabetic patients is more than double that of nondiabetic patients [20]. Uncontrolled chronic hyperglycemia with mitochondrial bioenergetics deficit and increased oxidative stress led to an inadequate physiological reserve in the myocardium when challenged by MI/R insult [21–24]. Diabetes also attenuates the endogenous antiapoptosis signaling pathways, thus invalidating powerful therapeutic programs, such as ischemic pre- and postconditioning [25–27]. Therefore, mitochondrial dysfunction and signaling events characteristic of apoptosis are responsible for the exacerbated cardiac ischemic injury in diabetic patients [28, 29]. The activation of prosurvival signaling cascades, such as PI3K/Akt, shows cardioprotective effects through recruitment of antiapoptotic proteins during MI/R in nondiabetes animals [30]. Consistent with our previous studies, diabetes mellitus markedly impairs the PI3K/Akt pathway and exacerbates MI/R injury [31, 32].

Many observations showed that HDACs inhibitor promotes an increase in Akt phosphorylation in different type of cells [33, 34]. HDACs inhibitor was found to protect brain white matter injury by activating the PI3K/Akt signaling pathway in neurons [34]. In fact, in Akt deleted mice, protective effects of TSA administration in reducing infarct size were blocked [35].

It has been best characterized that Forkhead box O3 (Foxo3a) plays a crucial role in regulating cell survival, oxidative stress resistance, and apoptosis. Foxo3a has been extensively studied in myocardial I/R injury [36, 37]. Phosphorylation of Foxo3a by serine/threonine kinase Akt results in nucleus exclusion and cytoplasm relocalization [38]. Recent work in mouse tissues demonstrated that the endogenous HDACs inhibitor caused an increased expression of Foxo3a [39]. It is worth noting that Foxo3a has been shown to modulate proapoptotic pathway through binding to consensus sequence of Bim promoter region and induce Bim expression [40].

Trichostatin A (TSA), a class I/II HDACs inhibitor [41], could protect the myocardium from I/R injury in nondiabetic rats [35]. Therefore, we aimed to investigate whether TSA elicits cardioprotective effect in an in vivo model of regional I/R in diabetic rat and in vitro model of hypoxia/reoxygenation (H/R) under hyperglycemia. Furthermore, we also detected whether the activation of Akt by TSA pretreatment could increase phosphorylation of Foxo3a.

## 2. Materials and Methods

### 2.1. Animals

Ninety Male Sprague-Dawley (SD) rats (250–280 g) were purchased from HFK bioscience (Beijing, China). Rats were housed in an environment with a maintained temperature of 22°C, relative humidity of 50 ± 15%, and a 12-hour light/12-hour dark cycle. All rats had free access to standard chow and water, ad libitum. All animal procedures received the approval of the Wuhan University Animal Care and Use Committee and followed the Principles of Laboratory Animal Care and are in accordance with the Guide for the Care and Use of Laboratory Animals by the National Institutes of Health (NIH Publication Number 80-23, revised 1996).

### 2.2. Reagents

Streptozotocin (STZ), triphenyl tetrazolium chloride (TTC), and Evans blue (EB) were obtained from Sigma Chemical Co. (MO, USA). TSA, the Akt inhibitor MK-2206, and the PI3K/Akt inhibitor wortmannin were purchased from Selleck Chemicals Co. (Texas, USA). Dulbecco's modified Eagle's medium (DMEM) and fetal bovine serum (FBS) were purchased from Gibco Laboratories (Grand Island, NY, USA). Antibodies directed against Akt, p-Akt (ser473), Foxo3a, p-Foxo3a (ser253), caspase-3, and GAPDH were obtained from Cell Signaling Technology (CST, Beverly, CA, USA). Primary antibodies against Bim, Bax, Bcl-2, cytochrome C (cytc), Lamin b1, acetyl-histone 3 (Ac-H3), acetyl-histone 4 (Ac-H4), histone 3 (H3), and histone 4 (H4) were obtained from Abcam (Cambridge, MA, USA).

### 2.3. Diabetes Induction

After 5 days of acclimation to the environment, rats were starved for 12 hours for diabetes induction. Diabetes was induced by single intraperitoneal (ip) injection of STZ (65 mg/kg). STZ was dissolved in 0.1 mol/L sodium citrate buffer (pH = 4.5). Control rats were injected with equal volume of buffer (0.1 mol/L sodium citrate buffer, pH 4.5) after an identical starvation process. Three days after STZ administration, blood samples were obtained from tail vein to evaluate the blood glucose, using Onetouch ultra glucometer (Johnson & Johnson, USA). If the fasting blood glucose was ≥16.7 mmol/L for at least three samples, the rats were considered to have diabetes.

### 2.4. Groups and Experimental Protocols

**Figure 1:**
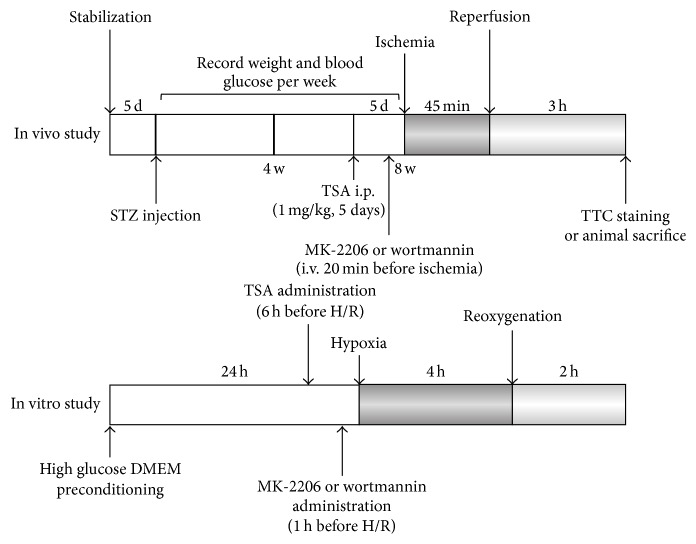
Experimental protocols. Diagram showing the experimental procedures including diabetes induction, ischemia/reperfusion model, TSA and MK-2206 administration, and sacrifice end point. Eight weeks after STZ injection, rats were subjected to 45 min of coronary artery occlusion followed by 3 h of reperfusion. TSA or DMSO was administrated at 48, 24, and 1 h before surgery. MK-2206, wortmannin, or vehicle was applied intravenously 20 minutes before ischemia. TSA: HDAC inhibitor; MK-2206: Akt inhibitor.

For the in vivo study, 8 weeks after STZ injection, diabetic and normal rats were randomized into four groups: (1) sham operated normal rats (NS), (2) normal rats subjected to I/R insult (NIR), (3) sham operated diabetic rats (DS), and (4) diabetic rats subjected to I/R insult (DIR). To gain a deeper insight into the prosurvival effect of HDACs inhibition, different interventions were preformed among diabetic rats: (1) diabetic rats subjected to I/R insult (DIR), (2) DIR rats pretreated with TSA (DIR+TSA), (3) DIR rats copretreated with TSA and MK-2206 (DIR+TSA+MK), and (4) DIR rats copretreated with TSA and wortmannin (DIR+TSA+Wor). TSA (1 mg/kg) or vehicle (DMSO, 1‰) was intraperitoneally given for 5 days before MI/R. MK-2206 (300 *μ*g/kg), wortmannin (15 ug/kg), or vehicle (DMSO, 1‰, 1 mL) was administered intravenously (femoral vein) 20 min before coronary ligation was performed. For the in vitro study, H9C2 cardiomyocytes were randomly assigned to seven groups: (1) low glucose control (LG-Control, 5.5 mM), (2) low glucose culture followed by H/R (LG-HR), (3) high glucose control (HG-control, 33 mM), (4) cells exposed to high glucose followed by H/R (HGHR), (5) HGHR cells pretreated with TSA (HGHR+TSA), (6) HGHR cells copretreated with TSA and Akt inhibitor (HGHR+TSA+MK), and (7) HGHR cells copretreated with TSA and PI3K/Akt inhibitor (HGHR+TSA+Wor). H9C2 cardiomyocytes were cultured in low glucose DMED with 10% FBS at 37°C in a humidified atmosphere of 10% CO_2_ and exposed to HG medium for 24 h followed by 4 h of hypoxia (94% N_2_ and 5% CO_2_) and 2 h reoxygenation. TSA (200 nM) or vehicle was administered 6 h before H/R. MK-2206 (300 nM), wortmannin (100 nM), or vehicle was given 1 h before hypoxia. The experimental protocol is depicted schematically in Figure 1.

### 2.5. Myocardial Ischemia and Reperfusion Model

8 weeks after diabetes induction, rats were anesthetized by pentobarbital sodium (50 mg/kg ip) and subjected to tracheotomy and artificial ventilation. Electrocardiogram and heart rate (HR) were continuously monitored by an electrophysiolograph (BioPAC, MH150, USA). Femoral vein intravenous infusion access had been established for drug or saline administration. The heart was exposed by a fifth-intercostal space thoracotomy and followed by pericardiotomy. A 6-0 silk suture was placed around the left anterior descending artery (LAD) between the first branch of the LAD and the left atrium. A small soft silicon tube was placed between the silk suture and the LAD. The ligature was tightened to occlude the LAD for 45 min. Successful MI/R model was verified by visual inspection of the LV apex for myocardial blanching and ST elevation on the surface electrocardiogram. After 45 min of ischemia, the slipknot was released to perform reperfusion for 3 h. The sham group received the same surgical procedures except for LAD occlusion. The animals were sacrificed after MI/R and part of the cardiac apex was rapidly removed for further analysis. Blood samples were obtained and centrifuged to obtain supernatant for future experiments.

### 2.6. HDACs Activity Assay

Nuclear extracts from the myocardium or H9C2 cells were suspended in ddH_2_O. After HDACs assay buffer addition, a colorimetric substrate comprising an acetylated lysine side chain was added and incubated for 1 h at 37°C. The reaction was stopped by adding lysine developer and then incubated for another 30 min. The lysine developer produces a chromophore that can be analyzed easily by an enzyme linked immunosorbent assay (ELISA) plate reader (Perkin Elmer Co. USA) at 400 nm. Results were expressed as relative OD values/*μ*g (% of sham or control ± SEM.).

### 2.7. Left Ventricle Function

Invasive hemodynamic measurements were performed to evaluate I/R induced cardiac dysfunction. A saline-filled catheter was inserted into the left ventricle via an incision on the right common carotid artery. The catheter was connected to a pressure transducer (Yixinda, Shenzhen, China). Left ventricular systolic pressure (LVSP) and maximal rates of increase and decrease in LVSP (±*dp/dt*max) were continuously monitored by an electrophysiolograph (BioPAC). Statistical analysis was performed by AcqKnowledge 4.0 software.

### 2.8. Determination of Myocardial Infarct Size

Six rats of each group were sacrificed to assess the sizes of the infarct area (IA), area at risk (AAR), and left ventricle (LV). Rats were administrated with heparin (1 U/g); the left coronary artery ligature was retired and 2 mL of 2% Evans Blue dye (Sigma, USA) was injected into the aorta immediately after 3 hours of reperfusion. Animals were sacrificed, and their hearts were excised and frozen at −70°C after washing with phosphate buffer saline (PBS). Hearts were sliced into 2 mm thick sections, perpendicular to the base-apex, and incubated with freshly prepared 1% TTC solution (pH 7.4) for 10 minutes at 37°C. The viable part was stained red by TTC, while the infarct portion remained pale. The infarct size was determined by an image analysis system (Image-Pro Plus 3.0; Media Cybernetics, MA). Two scientists scored the slides independently to ensure reliability of the results. The percentage of area at risk versus left ventricle (AAR/LV × 100%) and infarct area versus area at risk (IA/AAR × 100%) were calculated.

### 2.9. Measurement of Lactate Dehydrogenase and Creatine Kinase-MB Activities in Serum

In vivo, arterial blood samples were collected at the end of reperfusion and centrifuged at 2000 rpm for 10 minutes to collect the serum. In vitro, the supernatant was collected to measure the effluent LDH after H/R injury. Levels of cardiovascular biomarkers including creatine kinase-MB (CK-MB) and lactate dehydrogenase (LDH) were detected using a commercially available kit (Jiancheng Bioengineering, China).

### 2.10. Electron Microscopy

We use transmission electron microscopy to observe sarcomere and mitochondria ultrastructures. Approximately 2 mm^3^ tissue from the left ventricle was removed, fixed, and embedded in resin. Thin section slides of 1-2 *μ*m were prepared and oriented for longitudinal sectional views to get a better sight of the mitochondria and sarcomeres. The slides were stained, viewed, and photographed under a transmission electron microscope (HITACHI, HT7700) at ×7,000 (7K) magnification.

### 2.11. Determination of Apoptosis

In vivo study: the myocardial apoptosis rate was determined using an in situ cardiomyocyte terminal deoxynucleotidyl nick-end labeling assay (TUNEL). Left ventricular apical was quickly removed and incubated with 4% paraformaldehyde overnight at room temperature after reperfusion. Fluorescein-dUTP was added and nuclei were counterstained with 4′,6-diamidino-2-phenylindole (DAPI), according to the manufacturer's protocol (Roche, Indianapolis, USA). Samples were visualized using a laser scanning confocal microscope (Olympus, FV1000). Ten fields were chosen randomly for each sample and the apoptosis index was determined by dividing the number of positive-staining nuclei by the total number of nuclei in a given view field. Caspase-3 is a pivotal mediator of apoptosis; caspase-3 expression was performed to assess myocardial apoptosis specifically. In vitro study: after H/R, cells were collected and resuspended in binding buffer and incubated with fluorescein isothiocyanate- (FITC-) conjugated annexin V and propidium Iodide (PI) for 10 minutes in the dark. Cellular fluorescence was measured using a FACSCalibur instrument (BD Biosciences, USA). The data obtained from the cell population were analyzed using Cell Quest Pro software (BD Biosciences, USA).

### 2.12. Cell Viability Assay

Cell viability was determined by cell counting kit-8 (Dojindo, Kumamoto, Kyushu, Japan) in 96-well plates. H9C2 cells were cultured in 6-well plates. The CCK-8 solution was add to each well after the treatments and the plates were incubated for 3 h. The absorbance at 450 nm was measured using a microplate reader. The mean optical density (OD) of 6 wells in each group was used to calculate the percentage of cell viability.

### 2.13. ΔΨ*m* Measurement

JC-1 staining was preformed to measure ΔΨ*m* in H9C2 cells. Cells were incubated with JC-1 for 20 min at 37°C after H/R injury. The red/green fluorescence was detected with a microplate reader (Tecan Infinite 200). The wavelengths of excitation green fluorescence (monomeric form) of JC-1 were 514 nm, and wavelengths of 585 nm were used to detect the aggregated form of JC-1.

### 2.14. Mitochondrial Permeability Transition Pore (mPTP) in Cardiomyocytes

To examine the mPTP opening, H9C2 cardiomyocytes were measured with the mPTP fluorescence assay kit from Genmed Scientifics Inc. (Arlington, MA, USA). H9C2 cells were loaded with calcein-acetoxymethyl-ester (calcein-AM) (0.25 mM) and 8 mM cobalt chloride at 37°C for 20 min. Fluorescence intensity was observed by fluorescence microscope (Olympus, Bx 50-FLA) at 488 nm excitation and 525 nm emission. Results were represented as relative fluorescence intensity.

### 2.15. Western Blotting

After reperfusion, hearts were removed rapidly and frozen in liquid nitrogen. Ventricle tissue was sampled (*n* = 6 rats/group) and homogenized with a mixture of RIPA lysis buffer, PMSF (Beyotime, China), and a protease inhibitor cocktail (Roche, USA). The samples were homogenized and centrifuged at 12 × 10^3^*g* for 15 minutes at 4°C. Supernatants were collected and total protein concentration was determined by using a BCA kit (Beyotime). Equivalent amounts of proteins (20–50 *μ*g) were separated on a 10% SDS polyacrylamide gel and electrotransferred onto a PVDF membrane. The PVDF membrane was blocked with 5% bovine serum albumin (BSA, Sigma) for 1 hour at room temperature and then incubated with primary antibodies (1 : 1000) diluted in 5% bovine serum albumin (BSA), respectively, with gentle agitation overnight at 4°C. The membranes were washed three times in Tris Buffered Saline with Tween-20 (TBST) for 7 minutes each and incubated with secondary antibody (1 : 10000 goat anti-rabbit IgG, Cell Signaling Technology, USA) for 1 hour at room temperature. Signals were detected using a fluorescence imaging scanner (Odyssey, Germany) and optical densities were quantified using the Odyssey Image Analysis Software.

### 2.16. Statistical Analysis

All data were expressed as means ± SEM. Statistical analyses were performed using one-way or two-way ANOVA, followed by the Tukey HSD test. Statistical analysis was performed using Graphpad Prism 6.0 for Windows (GraphPad Software, USA). Statistical significance was accepted at *p* < 0.05.

## 3. Results

### 3.1. Diabetes Per Se and MI/R Injury Both Elevated HDACs Activity and Increased Infarct Size in Rat Hearts

**Figure 2:**
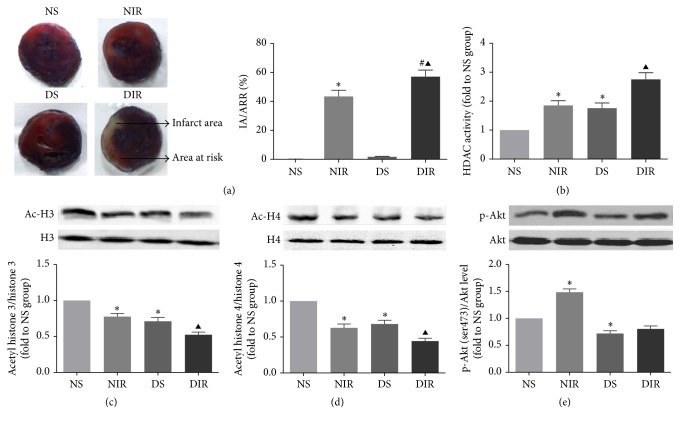
Myocardial infarct size and HDAC activity assessment in diabetic and normal rats. (a) Infarct area versus area at risk (IA/AAR × 100%). (b) Effect of MI/R and diabetes per se on total HDAC activity in myocardium. (c, d) Western blot analysis of myocardium acetylated histones H3 and H4 (Ac-H3, Ac-H4), with histones 3 or 4 (H3, H4) serving as the loading control. (e) p-Akt (ser473) and Akt protein expression in myocardium. All the results are presented as mean ± SEM, *n* = 6/group. ^*∗*^*p* < 0.05 versus NS group. ^▲^*p* < 0.05 versus DS group. ^#^*p* < 0.05 versus NIR group.

As shown in Figure 2(a), the myocardium infarct size of diabetic rats was significantly increased compared with the normal group (NIR versus DIR, *p* < 0.05). Total HDACs activity and specific HDACs substrates (Ac-H3 and Ac-H4) of the myocardium were measured to further investigate the relationship between HDACs activity and myocardial injury (Figure 2(b)). MI/R injury significantly elevated HDACs activity and reduced Ac-H3/H3 and Ac-H4/H4 ratios (Figures 2(c) and 2(d)) in both normal and diabetic rats (NS versus NIR, DS versus DIR, all *p* < 0.05). The total HDACs activity was increased in diabetic rats as compared with the normal rats, which was further confirmed by decreased ratios of Ac-H3/H3 and Ac-H4/H4 (NS versus DS, *p* < 0.05). Next we used immunoblotting to evaluate phosphorylation level of Akt in diabetic and nondiabetic rats, in an attempt to explain why diabetes aggravates MI/R injury. The results showed that MI/R injury elicited a remarkable rise in p-Akt levels in the NIR group compared with NS group; however, this protective reaction was attenuated by diabetes mellitus (DS versus DIR, *p* < 0.05, Figure 2(e)).

### 3.2. HDACs Inhibition Attenuated Myocardial Injury in Diabetic Rats

**Figure 3:**
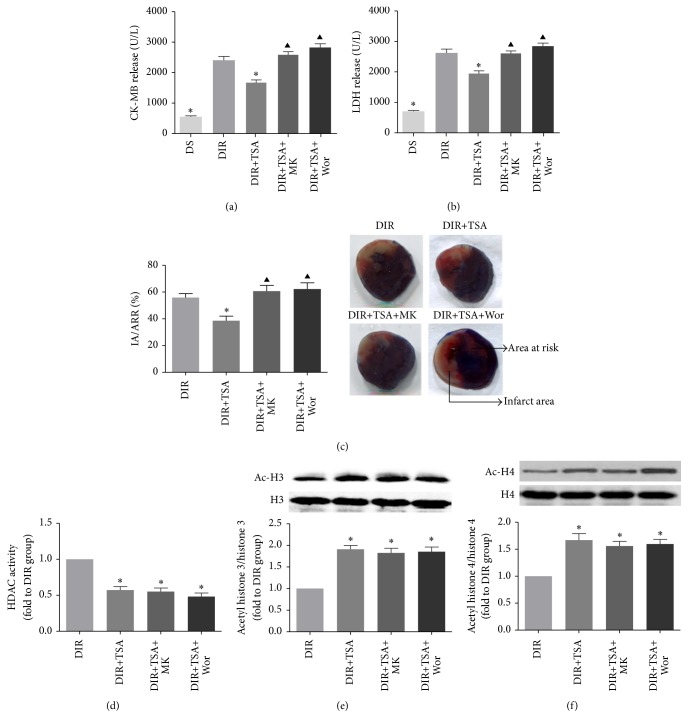
The HDAC inhibitor attenuated MI/R injury in diabetic hearts. Serum levels of myocardial injury marker CK-MB (a) and LDH (b). (a) Infarct area versus area at risk (IA/AAR × 100%). Blue-stained areas represent the nonischemic part; red-stained areas represent the area at risk and pale areas indicate myocardial infarction. Area at risk and infarct area were pointed out by arrows. HDACs activity (d) and acetylated level of histone 3 (e) and histone 4 (f). All the results are presented as mean ± SEM, *n* = 6/group. ^*∗*^*p* < 0.05 versus DIR group. ^▲^*p* < 0.05 versus DIR+TSA group.

We further evaluated the effect of TSA on MI/R injury. Serum CK-MB and LDH levels were significantly increased in diabetic rats compared with age-matched control rats (DS versus DIR, *p* < 0.05, Figures 3(a) and 3(b)). However, TSA pretreatment alleviated MI/R induced cardiomyocyte injury in diabetic rats, as demonstrated by the lower CK-MB and LDH levels (DIR versus DIR+TSA, *p* < 0.05, Figures 3(a) and 3(b)). TSA pretreatment markedly reduced the infract size in diabetic rats (55.9% ± 2.97% versus 38.6% ± 3.45%, *p* < 0.05, Figure 3(c)). Hemodynamic parameters were measured to evaluate the left ventricle function (Table 1). TSA treatment significantly increased +*dp*/*dt*, −*dp*/*dt*, and LVSP after 3 h of reperfusion (DIR versus DIR+TSA, *p* < 0.05). Notably, copretreatment with MK-2206 or wortmannin inhibited the TSA-induced protective effects on LV function, infarct size, and release of CK-MB and LDH (DIR+TSA versus DIR+TSA+MK or DIR+TSA+Wor, all *p* < 0.05). TSA was proved to be an effective HDACs inhibitor in present study; pretreatment of TSA for 5 days before surgery in diabetic rats significantly reduced HDACs activity and increased the level of acetylated H3 and H4 (DIR versus DIR+TSA, all *p* < 0.05, Figures 3(d), 3(e), and 3(f)) in myocardium.

### 3.3. TSA Pretreatment Attenuated I/R Induced Apoptosis

**Figure 4:**
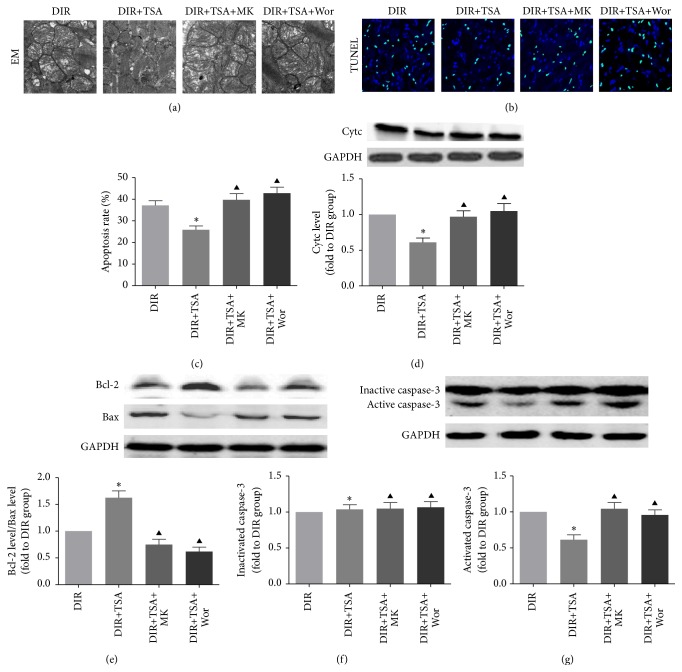
HDAC inhibition attenuated mitochondria-associated apoptosis in diabetic rats. (a) Representative electron micrographs of each group (×7000). (b) Representative TUNEL staining images of each group. (c) Quantitative analysis of the apoptosis rate in each group. (d, e) Representative immune blot images and assessment of the cytochrome C (cytc), Bcl-2, and Bax level in each group. (f, g) Representative immune blot images and assessment of activated and inactivated caspase-3 in cardiac tissue. All results are presented as the mean ± SEM, *n* = 6/group. ^*∗*^*p* < 0.05 versus DIR group. ^▲^*p* < 0.05 versus DIR+TSA group.

To examine whether TSA pretreatment could alleviate I/R induced myocardial cells apoptosis in diabetic rats, we evaluated the apoptosis rate by a TUNEL assay (Figures 4(b) and 4(c)), further confirmed by the expression of mitochondrial apoptosis-associated proteins caspase-3 (Figures 4(f) and 4(g)), cytc (Figure 4(d)), and Bcl-2/Bax ratio of each group (Figure 4(e)). There were no changes in total (inactivated) caspase-3 levels in the 4 groups. TSA pretreatment significantly decreased the activated caspase-3, the number of TUNEL-positive cells, and cytc expression; however, Bcl-2/Bax ratio was upregulated (DIR+TSA versus DIR, all *p* < 0.05). Copretreatment with MK-2206 or wortmannin in diabetic rats before MI/R significantly elevated expression of activated caspase-3 and increased the number of TUNEL-positive cells compared with that observed in the DIR+TSA group (both *p* < 0.05). Further study found that the effects of TSA on Bcl-2/Bax ratio and cytc expression were reversed by MK-2206 or wortmannin administration (DIR+TSA versus DIR+TSA+MK or DIR+TSA+Wor group, all *p* < 0.05). The ultrastructure changes of myocardium were observed under an electron microscope. Representative electron micrographs of each group are shown in Figure 4(a). Vacuolar degeneration and cristae fragmentation of myocardial mitochondria caused by MI/R were observed. However, TSA pretreatment mitigated myocardial mitochondrial swelling and attenuated the cellular edema.

### 3.4. Akt and Foxo3a Play a Crucial Role in the Protective Effect of TSA

**Figure 5:**
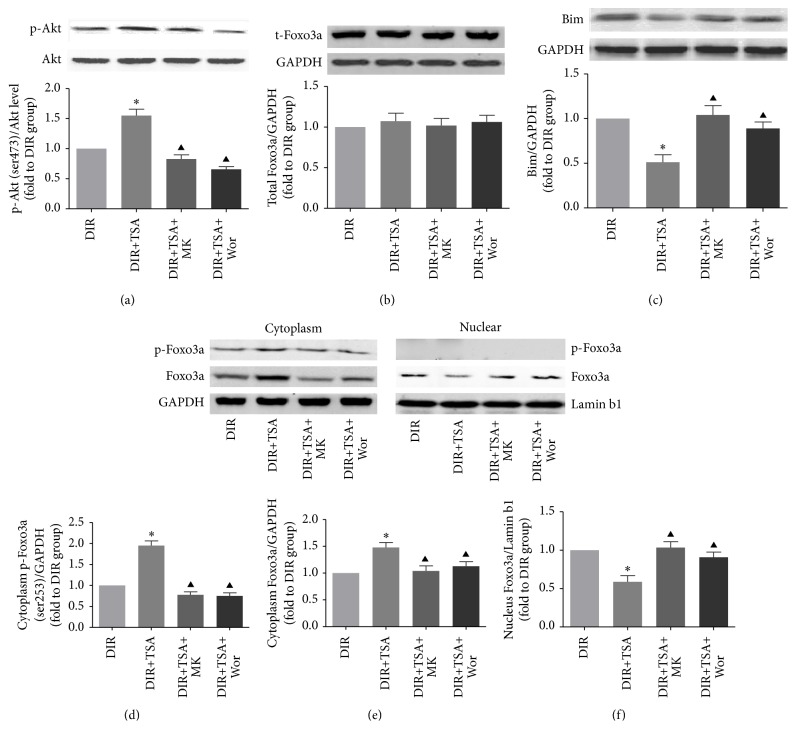
Expressions of p-Akt, p-Foxo3a, and Bim. Representative myocardial p-Akt, total-Akt (a), total-Foxo3a (b), Bim (c), cytoplasm p-Foxo3a (d), cytoplasm Foxo3a (e), and nuclear Foxo3a (f) Western blots images and protein levels analysis. GAPDH or Lamin b1 served as the loading control. All values are presented as the mean ± SEM, *n* = 6/group. ^*∗*^*p* < 0.05 versus DIR group. ^▲^*p* < 0.05 versus DIR+TSA group.

The expressions of Akt/p-Akt, Foxo3a/p-Foxo3a, and Bim were determined by Western blotting. Notably, TSA administration increased the expression of p-Akt in diabetic rats when exposed to MI/R insult (DIR versus DIR+TSA, *p* < 0.05), while the p-Akt level was markedly decreased after an Akt inhibitor was applied (DIR+TSA versus DIR+TSA+MK or DIR+TSA+Wor, *p* < 0.05, Figure 5(a)). As total-Foxo3a level remained unchanged in each group (Figure 5(b)), we measured p-Foxo3a and Foxo3a levels in both cytoplasm and nucleus lysate (Figures 5(d), 5(e), and 5(f)). Immunoblotting results showed that TSA administration significantly increased phosphorylation level of p-Foxo3a and Foxo3a in cytoplasm. However, copretreatment with the Akt inhibitor decreased the expression of p-Foxo3a and Foxo3a in cytoplasm (DIR+TSA versus DIR+TSA+MK or DIR+TSA+Wor, all *p* < 0.05). These results suggested that phosphorylation of Foxo3a leads to nuclear exclusion and cytoplasm relocalization. Next, we measured the expression of proapoptosis protein Bim. Researchers have shown that Foxo3a binds to a consensus sequence in the Bim promoter region and activates Bim transcription [42]. As expected, Bim expression was downregulated by the HDACs inhibitor in diabetic rats (DIR versus DIR+TSA, *p* < 0.05, Figure 5(c)). However, the suppression of Bim by TSA pretreatment was abolished by MK-2206 or wortmannin administration (DIR+TSA versus DIR+TSA+MK or DIR+TSA+Wor, all *p* < 0.05).

### 3.5. High Glucose Induced Cardiomyocytes Apoptosis and Prosurvival Akt Pathway Deactivation When Exposed to Hypoxia/Reoxygenation Injury

**Figure 6:**
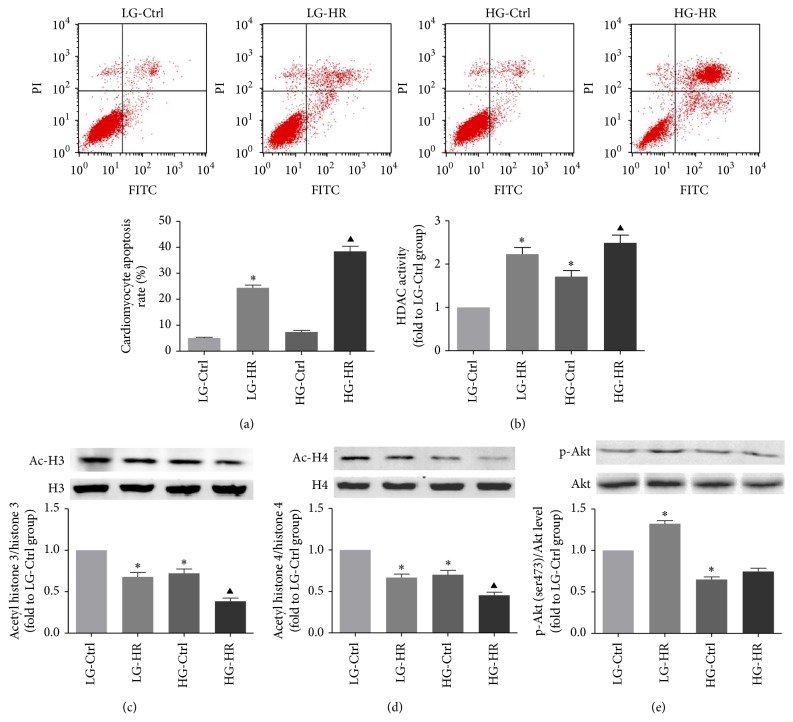
Apoptosis rate and HDAC activity assessment in H9C2 cells. Apoptosis of H9C2 cells was assessed by flow cytometry. (b) Total HDAC activity of cardiomyocytes in each group. (c, d) Western blot analysis of cardiomyocyte Ac-H3/H3 and Ac-H4/H4. (e) p-Akt (ser473)/Akt ratio in H9C2 cells. All values are presented as the mean ± SEM, *n* = 6/group. ^*∗*^*p* < 0.05 versus LG-Ctrl group. ^▲^*p* < 0.05 versus HG-Ctrl group.

The representative flow cytometry images of LG-Control, LG-HR, HG-Control, and HGHR groups are shown in Figure 6. H9C2 cells exposed to high glucose for 24 h and followed by H/R showed a marked increase in apoptosis compared with the LG-HR group (Figure 6(a)). To further evaluate the relationship between HDACs activity and myocardial injury, total HDACs activity (Figure 6(b)) and the levels of Ac-H3/H3 (Figure 6(c)) and Ac-H4/H4 (Figure 6(d)) in H9C2 cells were measured. HDACs activity of H9C2 cells lysate was elevated after 24 h exposure to high glucose, while Ac-H3/H3 and Ac-H4/H4 ratios were decreased. H/R injury also elevated the total HDACs activity, which was further confirmed by decreased expression of Ac-H3/H4 and Ac-H4/H4 ratios (LG-Ctrl versus LG-HR; HG-Ctrl versus HGHR; all *p* < 0.05). Furthermore, we measured the effect of HG on Akt activation in H9C2 cells. Immunoblotting showed that H/R injury elicited a remarkable increase of p-Akt levels in the LG-HR group (LG-Ctrl versus LG-HR, *p* < 0.05). However, high glucose attenuated this spontaneous protective reaction (HG-Ctrl versus HGHR, *p* < 0.05, Figure 6(e)).

### 3.6. The HDACs Inhibitor Induced Protection of Cardiomyocytes Exposed to HGHR Injury

**Figure 7:**
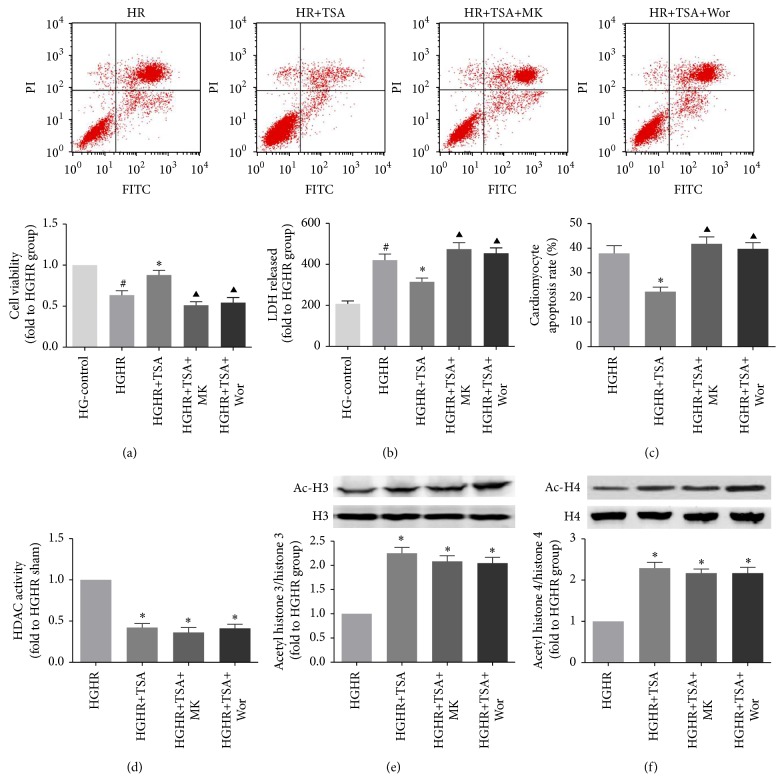
TSA administration has a protective effect on cardiomyocytes. Apoptosis in H9C2 cardiomyocytes was assessed by cell viability (a) and LDH leakage (b) and apoptosis rate (c) in each group. Cell lysate HDAC activity of each group was measured (d, e, f). Representative Ac-H3/H3 and Ac-H4/H4 Western blots images and protein levels analysis. GAPDH served as the loading control. All values are presented as the mean ± SEM, *n* = 6/group. ^#^*p* < 0.05 versus HG-Control group. ^*∗*^*p* < 0.05 versus HGHR group. ^▲^*p* < 0.05 versus HGHR+TSA group.

The representative flow cytometry images from the HGHR, HGHR+TSA, HGHR+TSA+MK, and HGHR+TSA+Wor groups were shown in Figure 7. H/R injury significantly decreased cell viability and increased LDH leakage (HG-Control versus HGHR, *p* < 0.05, Figures 7(a) and 7(b)), while TSA pretreatment attenuated cellular injury and reduced cell apoptosis, as demonstrated by a decreased cells apoptosis rate, reduced LDH leakage, and increased cell viability (HGHR versus HGHR+TSA, *p* < 0.05, Figures 7(a), 7(b), and 7(c)). Copretreatment with MK-2206 or wortmannin partly blocked the protective effects of the HDACs inhibitor (HGHR+TSA versus HGHR+TSA+MK or HGHR+TSA+Wor, *p* < 0.05). TSA was proved to be an effective HDACs inhibitor in H9C2 cardiomyocytes; administration of TSA 6 h before H/R in H9C2 cells significantly reduced HDACs activity and increased acetylated level of H3 and H4 (HGHR versus HGHR+TSA, all *p* < 0.05, Figures 7(d), 7(e), and 7(f)).

### 3.7. Effects of TSA Administration on mPTP

**Figure 8:**
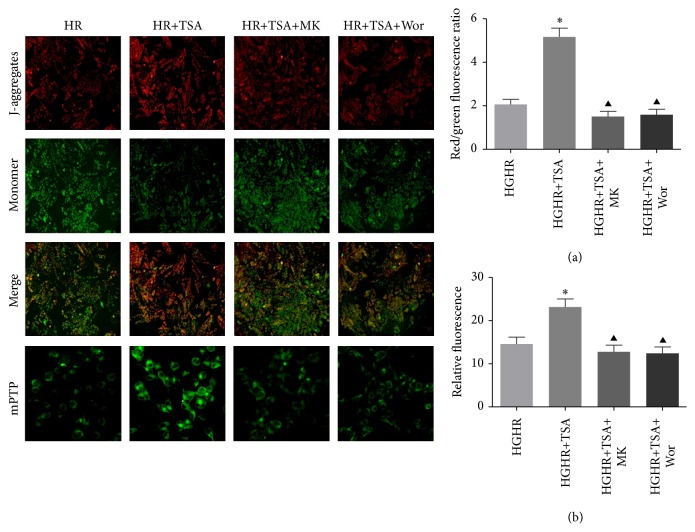
Effect of HDAC inhibition on mitochondrial membrane potential and integrity of mitochondrial permeability transition pore (mPTP). Representative fluorescent images and quantitative analysis in each group. (a) Red fluorescence represented JC-1 aggregation on the mitochondria membrane, reflecting the mitochondrial membrane potential (ΔΨ*m*). Green emission of the dye represented the monomeric form of JC-1 after mitochondrial membrane depolarization. (b) Fluorescent images of the cells show the change in integrity of mPTP. Reduced fluorescence in cells indicate opening of mPTP. All values are presented as the mean ± SEM, *n* = 6/group. ^*∗*^*p* < 0.05 versus HGHR group. ^▲^*p* < 0.05 versus HGHR+TSA group.

Due to the property of calcein-AM and cobalt chloride, the fluorescence intensity of calcein-AM following treatment with cobalt chloride can reflect the extent of mPTP opening. Compared with the HGHR, integrity of mPTP was altered significantly in TSA treated cells which was evident from increased calcein fluorescence (Figure 8(b)). However, cotreatment of MK-2206 or wortmannin demonstrated a significant reduced fluorescence level compared with TSA group (Figure 8(b)). These results indicated that TSA decreased the degree of mPTP opening in response to H/R induced injury under high glucose condition.

### 3.8. TSA Suppressed ΔΨ*m* Dissipation in H9C2 Cells

Mitochondrial membrane potential (ΔΨ*m*) was assessed by JC-1 staining (Figure 8(a)). JC-1 aggregates mitochondria and shows red fluorescence under normal conditions. Exposure of H9C2 cells to high glucose for 24 h, followed by H/R resulted in dissipation of ΔΨ*m*, which was shown as increased green fluorescence after JC-1 staining. Pretreatment with TSA could moderate the ΔΨ*m* dissipation by HGHR injury, as demonstrated by increased red/green fluorescence ratio, which indicated the protective effect of TSA (HGHR versus HGHR+TSA, *p* < 0.05). Compared with the HGHR+TSA group, cells copretreated with MK-2206 or wortmannin showed aggravated dissipation of ΔΨ*m* (HGHR+TSA+MK or HGHR+TSA+Wor versus HGHR+TSA, *p* < 0.05).

### 3.9. Inhibition of HDACs Elevated the Phosphorylation Levels of Akt and Its Downstream Effectors in H9C2 Cells Exposed to HG and HR Injury

**Figure 9:**
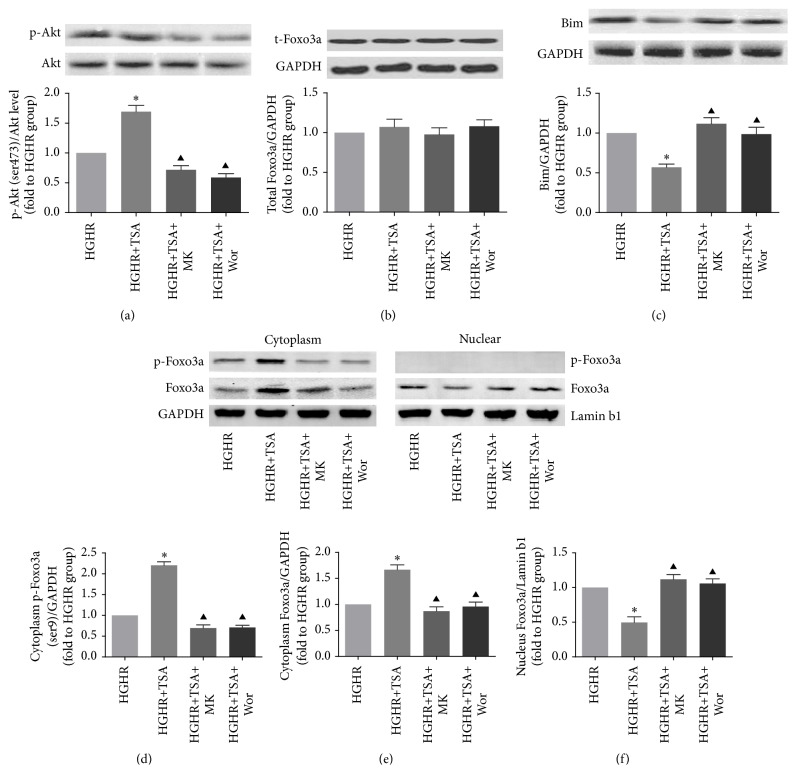
Expression of p-Akt/Akt, p-Foxo3a/Foxo3a, and Bim. Cardiomyocyte p-Akt, total-Akt (a), total-Foxo3a (b), Bim (c), cytoplasm p-Foxo3a (d), cytoplasm Foxo3a (e), and nuclear Foxo3a (f) Western blots images and protein levels analysis. GAPDH or Lamin b1 served as the loading control. ^*∗*^*p* < 0.05 versus HGHR group. ^▲^*p* < 0.05 versus HGHR+TSA group.

TSA administration before HGHR injury induced a considerable increase in p-Akt, while Bim expression was suppressed (HGHR versus HGHR+TSA, all *p* < 0.05, Figures 9(a) and 9(c)). As total Foxo3a remained unchanged in each group (Figure 8(b)), we measured p-Foxo3a and Foxo3a levels in both cytoplasm and nuclear lysate (Figures 9(d), 9(e), and 9(f)). Immunoblotting results showed that TSA administration significantly increased phosphorylation level of p-Foxo3a and Foxo3a in cytoplasm. Additionally, copretreatment with the Akt inhibitor decreased the expression of p-Foxo3a/Foxo3a in cytoplasm (DIR+TSA versus DIR+TSA+MK or DIR+TSA+Wor, all *p* < 0.05, Figure 9(b)). These results showed that phosphorylation of Foxo3a may lead to nuclear exclusion and cytoplasm relocalization.

## 4. Discussion

To the best of our knowledge, this is the first study to demonstrate that the HDACs inhibition confers cardioprotection against MI/R injury in diabetic rats that were more vulnerable to ischemic injury. Reversible acetylation of lysine in histone or nonhistone proteins by HDACs is a key mechanism for signal modification and precise gene transcriptional regulation, which are essential for cardiovascular pathophysiology [43, 44]. Accumulated studies have revealed that diabetes mellitus causes increased HDACs activity in the liver, pancreas [45], and kidney [46]. In line with these researches, our study demonstrated that cardiac HDACs activity is elevated by both the duration of the hyperglycemic state and MI/R injury.

TSA is well established to inhibit class I/II HDACs activity by blocking the active site of HDACs [41]. TSA shows certain cardioprotective effects in nondiabetic heart I/R Injury [47, 48]. In our present study, we found for the first time that both MI/R injury and diabetes per se could increase total HDACs activity in rat heart; furthermore, class I/II HDACs inhibitor TSA triggered protective effects against MI/R and H/R injury under diabetic conditions.

Recent studies indicated that inhibition of histone deacetylases is a promising drug target to confer protection against ischemic or hypoxic injury [13, 35, 49]; the molecule mechanisms that underlie the HDACs inhibition-induced cardioprotection still remain unknown [50]. Accumulated experimental data indicated that TSA activates PI3K/Akt signaling. Wang et al. reported that TSA transiently activated PI3K/Akt cell survival pathway and inhibition of this signaling pathway promoted TSA's effect on cell death and migration in ovarian cancer cells [34]. Furthermore, recent study showed TSA could significantly reduce the cerebral infarct volume during cerebral ischemia/reperfusion injury, which was achieved partly by activation of the PI3K/Akt signaling pathway via upgrading of p-Akt protein. Importantly, our results demonstrated that the selective inhibition of PI3K/Akt signaling pathways by wortmannin could weaken cardioprotective effect induced by TSA treatment dramatically, indicating the essential effect of PI3K/Akt pathways in mediating cardioprotection elicited by HDACs inhibition.

Loss of endogenous reactive Akt activation in the diabetic myocardium may help to explain why diabetic rats are more vulnerable to MI/R injury [51, 52]. We further investigated the role of downstream apoptosis-associated effectors of Akt in the TSA-induced protective effect. We found that TSA treatment caused an increased expression of phosphorylated Foxo3a in cytoplasm but a decreased expression of Foxo3a in nuclear; however these effects were suppressed by Akt inhibitor. Furthermore, we found that the decreased expression of nuclear p-Foxo3a was accompanied by a reduction of Bim expression. We concluded that phosphorylation of Foxo3a by Akt may result in nucleus exclusion and cytoplasm relocalization, thus deactivating the Foxo3a pathway [38, 53]. Notably, Foxo3a modulates proapoptotic pathways by binding to the consensus sequence of the Bim promoter region and inducing Bim expression [40]. In vivo and vitro experiments revealed that TSA administration induced Foxo3a's cytoplasmic relocation and downregulated Bim expression. Bim blocks stress signals transduced from the cytoplasm to mitochondria and triggers the Bax-mediated mitochondria apoptosis pathway [54]. Our result may help explain how TSA protected diabetic rats from the cardiac-damage induced by ischemia or hypoxia.

The survival and antiapoptosis effects of HDACs inhibition were closely associated with the reduction of the apoptosis rate of cardiomyocytes. Signaling events characteristic of apoptosis and mitochondrial dysfunction resulted in the diabetic myocardium being more vulnerable to I/R injury [28, 29]. Ischemic or hypoxic injury renders the mitochondrial membrane permeable and causes dissipation of the proton gradient across the mitochondrial membrane. One of the main events that trigger mitochondrial dysfunction is mPTP opening, with subsequent mitochondrial swelling [55]. Cytochrome C is located within the intermembrane space of mitochondria under normal conditions [56]. I/R injury results in the mPTP opening and cytochrome C release [57]. In the present study, class I/II HDACs inhibition mitigated the effects of MI/R on ΔΨ*m* loss, integrity of mPTP, and cytochrome C release. Pretreatment with a HDACs inhibitor before MI/R or H/R moderated the dissipation of ΔΨ*m* and protected integrity of mPTP by suppressing Bim expression, which mediates the mitochondrial apoptosis process by opening mPTP on the mitochondrial membrane [57].

Our observation shows that HDACs inhibition protects diabetic heart against I/R injury; however the roles of class I HDACs or class II HDACs in myocardial protection is still controversial. The study of Aune et al. demonstrated that class I HDACs inhibition elicits protection of contractile function following I/R, which is associated with increased expression of endogenous antioxidant enzymes [58]. Class II HDACs are abundantly expressed in the heart [59]. Study from Zhao et al. supports that class II serves as a potential target in MI/R injury through TSA inhibition [35]. Further studies need to be done to determine whether the selective inhibition of HDACs exerts cardiac protection from MI/R injury in diabetic heart. Pretreated diabetic rats with a HDACs inhibitor suppressed the excessive HDACs activity in the myocardium and conferred protection against MI/R injury. We believe that high levels of cardiac HDACs activity in diabetes exacerbate ischemic heart injury; however, the underlying mechanisms require further investigation.

## 5. Conclusions

Both duration of diabetes per se and MI/R injury increased cardiac HDACs activity. Suppression of HDACs activity triggered protective effects against MI/R and H/R injury under hyperglycemia conditions through Akt-modulated mitochondrial apoptotic pathways via Foxo3a/Bim.

## Figures and Tables

**Table 1:** Effects of TSA on LV hemodynamic parameters after MI/R.

Group/parameters	DIR	DIR+TSA	DIR+TSA+MK	DIR+TSA+Wor
LVDP (mmHg)	52.32 ± 2.78	72.47 ± 4.26^*∗*^	54.24 ± 3.29^▲^	48.11 ± 2.96^▲^
+*dp/dt*max (mmHg/s)	2361 ± 227.5	3582 ± 264.3^*∗*^	2564 ± 238.7^▲^	2371 ± 219.8^▲^
−*dp/dt*max (mmHg/s)	2574 ± 189.1	3672 ± 232.6^*∗*^	2658 ± 263.4^▲^	2021 ± 209.8^▲^

Results represent mean ± SEM. ^*∗*^*p* < 0.05 versus DIR; ^▲^*p* < 0.05 versus DIR+TSA; *n* = 9–12/group.
